# Assessment of HIV testing among young methamphetamine users in Muse, Northern Shan State, Myanmar

**DOI:** 10.1186/1471-2458-14-735

**Published:** 2014-07-21

**Authors:** Yu Mon Saw, Krishna C Poudel, Nang Pann Ei Kham, Nyein Chan, Jessica E Cope, Kyi Mar Wai, Soe Tun, Thu Nandar Saw

**Affiliations:** 1 Women Leaders Program to Promote Well-being in Asia, School of Health Sciences, Graduate School of Medicine, Nagoya University, 1-1-20, Daiko-minami, Higashi-ku, Nagoya 461-8673, Japan; 2 Department of Public Health, School of Public Health and Health Sciences, University of Massachusetts Amherst, Amherst, MA, USA; 3 Institute for Drug and Alcohol Studies, Virginia Commonwealth University, Richmond, Virginia, USA; 4 Department of Social Research, Defence Services Medical Research Centre, Tatkone Township, Nay Pyi Taw, Myanmar; 5 Department of Community and Global Health, Graduate School of Medicine, the University of Tokyo, Tokyo, Japan; 6 Department of Preventive and Social Medicine, the University of Medicine 2, Yangon, Myanmar

**Keywords:** HIV testing, Methamphetamine, Youth, Myanmar

## Abstract

**Background:**

Methamphetamine (MA) use has a strong correlation with risky sexual behaviors, and thus may be triggering the growing HIV epidemic in Myanmar. Although methamphetamine use is a serious public health concern, only a few studies have examined HIV testing among young drug users. This study aimed to examine how predisposing, enabling and need factors affect HIV testing among young MA users.

**Methods:**

A cross-sectional study was conducted from January to March 2013 in Muse city in the Northern Shan State of Myanmar. Using a respondent-driven sampling method, 776 MA users aged 18-24 years were recruited. The main outcome of interest was whether participants had ever been tested for HIV. Descriptive statistics and multivariate logistic regression were applied in this study.

**Results:**

Approximately 14.7% of young MA users had ever been tested for HIV. Significant positive predictors of HIV testing included predisposing factors such as being a female MA user, having had higher education, and currently living with one’s spouse/sexual partner. Significant enabling factors included being employed and having ever visited NGO clinics or met NGO workers. Significant need factors were having ever been diagnosed with an STI and having ever wanted to receive help to stop drug use.

**Conclusions:**

Predisposing, enabling and need factors were significant contributors affecting uptake of HIV testing among young MA users. Integrating HIV testing into STI treatment programs, alongside general expansion of HIV testing services may be effective in increasing HIV testing uptake among young MA users.

## Background

Globally, the use of amphetamine-type stimulants (ATS) including methamphetamines (MA) is a significant public health concern. In terms of usage, ATS are the second most commonly used group of stimulant substance, with MA the most prominent within this group [1,2]. In 2010, worldwide estimated prevalence for ATS use ranged between 0.3% to 1.2%, with the number of people aged 15– 64 years who had used a stimulant substance at least once reported to be between 14 to 52 million [2]. A growing body of evidence suggests that stimulant substance use has a strong association with risky sexual behaviors, which in turn can be correlated with HIV and other sexually transmitted infections [3,4]. Increases in the number of young MA users appear to have been mirrored by increasing numbers of young HIV-infected individuals [5].

HIV testing is a cornerstone of HIV case detection, prevention, care and treatment [6]. With the advent of more effective therapies in recent years, early diagnosis is important for optimizing treatment plans and forestalling the progression to AIDS and death. Furthermore, earlier awareness of seropositivity may lead to decreased future transmission of the virus, by shortening the period during which an asymptomatic HIV-infected individual could unknowingly spread the virus [6-8]. However, most young drug users have limited awareness of the negative health risks of their drug-related behaviors, such as HIV, as well as poor general knowledge and awareness of HIV infection [9,10].

In Myanmar, HIV infection mainly occurs among key affected populations, such as drug users, sex workers and their clients, and men who have sex with men (MSM). For example, HIV prevalence among 16-24 year old female sex workers, MSM and injecting drug users was 9.1%, 5.7% and 13.7% respectively in 2011 [11]. Since the use of ATS has dramatically increased in Myanmar over the past few decades, it has been suggested that this might have triggered the growing HIV epidemic [3,4,12,13]. Young people account for 14% of the population in Myanmar [14], and MA use and HIV prevalence is rising among these groups [15]. Therefore, it is important to encourage young drug users to be tested for HIV infection. Failing to provide adequate HIV prevention among the youth population could lead to large numbers of future adult AIDS cases, which could be devastating to the economy and supply of human resources in Myanmar. This study thus aimed to determine the rates and predictors of HIV testing among young MA users. This study applied the Gelberg-Andersen Behavioral Model for Vulnerable Populations to identify predictors of testing among young MA users [16]. This study hypothesized that young MA users are a vulnerable group due to their increased risk for engaging in substance misuse and risky sexual behaviors. Identification of predisposing, enabling and need factors associated with HIV testing will be helpful for the development of further strategies in HIV case detection, treatment and prevention programs among young MA users in Myanmar and South-east Asia in general.

## Methods

### Study design and participants

A cross-sectional study was conducted from January to March 2013 in Muse city in the Northern Shan State of Myanmar, located close to the China border. Within Myanmar, the majority of methamphetamine pill seizures are made near the production areas in the border towns of North Shan State [17]. The available seizure information suggests that there are a large number of groups involved in methamphetamine use, production, and trafficking with border cities in China. According to the Sentinel Surveillance in 2009, HIV prevalence among the drug using population in Muse city was 36.7% [11].

Study participants were recruited using the following criteria: 1) self-reported MA users aged between 18 to 24 years; 2) had used methamphetamine drugs in the last 6 months; 3) exhibited no withdrawal symptoms and not under the influence of drugs at the time of interview; and 4) able to read the Myanmar language. A total of 776 MA users’ information was collected.

### Data collection procedure

Data was collected in a private place using a self-administered computer program known as the computer-assisted survey instrument (CASI), which requested participants to complete surveys on a laptop computer. CASI has the capacity to improve data collection on sensitive behaviors, particularly among high-risk populations [18-20]. The participants were selected by respondent-driven sampling (RDS), which is used to recruit hard-to-access and hidden populations [21,22]. A total of eight leaders (four males and four females) acted as the first seeds. The main seeds were interviewed and requested to each recruit three other MA users to take part in this study. In the second wave, the newly recruited MA users were in turn requested to each recruit three more MA user friends after completing their interviews. Each participant was allowed to recruit up to three people to participate in the survey, in accordance with RDS recruitment procedure. Several studies have reported that equilibrium can be achieved after five to six waves of recruitment using RDS methods [23,24]. In this study, equilibrium was reached at wave six. All participants received compensation of 2000 kyats (approx. $2.5) for their time and transportation costs immediately after their interviews. They were eligible to receive a secondary incentive of 900 kyats (approx. $ 1.2) after the MA users whom they recruited participated in the study.

This study was approved by the Research Ethics Committee of the Graduate School of Medicine, University of Tokyo, Japan, and the Institutional Ethical Review Committee, Defense Services Medical Research Centre, Nay Pyi Daw, Myanmar. Before conducting each interview, participants were clearly informed of the purpose of this study and pertinent study procedures. If participants fully understood and decided to participate, they were requested to read about informed consent on the computer screen and click “agree to participate in this study” to answer the survey questions for their voluntary participation. They were also informed that they could skip answering any question they did not want to answer, and could withdraw from participation at any time during, or after the interview, without penalty. Confidentiality of the entire data set was maintained at all stages of data collection and analyses.

This study selected the variables suggested by the Behavioral Model from the previous studies [16,25-27]. The questionnaire of this study was first prepared in English. Then, English version questionnaire was translated to Myanmar language, and was reviewed by three Myanmar public health experts who are working on drug use and sexual behavior issues. Back-translation of the instrument from the Myanmar language to English was done to ensure semantic equivalence. The questionnaire was pre-tested among 40 MA users in October 2012 by the researchers. Finally, the questionnaire was modified based on the results of the pre-test to make it more understandable and easier for participants to answer.

Five supervisors (coupon managers) and nine MA peer CASI interview assistants supported the CASI interviews. The supervisors and CASI interview assistants attended a one-day training prior to conducting the survey to learn the study objectives and procedures, and the importance of maintaining the confidentiality of the participants’ information. The training was done using a field manual that was developed in the Myanmar language. The field supervisor also went along to the study sites and supervised the fieldwork to assure interview quality.

### Measures

#### Dependent variable

The dependent variable was whether or not the respondent had ever been tested for HIV, which was dichotomized as “0” for never tested and “1” as ever tested.

#### Independent variables

Potential predictors were selected using Gelberg and colleagues’ Behavioral Model for Vulnerable Populations [16], which posits that health-seeking behavior among vulnerable groups, such as young drug users, is a function of three categories of variables: predisposing, enabling, and need factors. Predisposing factors reflect that the individual’s characteristics can affect their access to health services, and were included in this study as socio-demographic characteristics: age, gender, marital status, education, ethnicity, and current living status. Enabling factors reflect the circumstances of individuals or families, as although people may want to use health services, they still need means of access [19]. Enabling factors included in this study were the occupational status of the individual, as well as whether the drug users had migrated from other parts of the country and whether they had ever visited non-governmental organization (NGO) clinics or met NGO workers. Migration status was considered to be an enabling factor due to specific impediments for migrants in accessing health services, such as legal, administrative [28,29] language [30,31] and cultural barriers [28,32]. This study considered in-country including the highlanders. However, migration across national borders, such as those with Thailand and China, was not included. Contact with NGOs was considered to be an enabling factor due to the potential opportunities they provide for drug users to access HIV risk assessment, education, and referral for testing and counseling [33].

Need factors reflect perceived health status, which provides the impetus for seeking healthcare. Two of the important need variables considered in this study were perceived HIV risk and whether participants had ever been diagnosed with a sexually transmitted infection (STI) [34,35]. The need factors thus included drug use behavior and treatment related variables such as having ever accessed drug treatment, ever shared injection equipment, used heroin in the last 6 months, ever drunk alcohol, ever wanted to receive help to stop drugs, and ever been convicted for a drug related offence.

### Statistical analysis

Data was analysed using the Statistical Package for the Social Sciences (SPSS) version 18 (SPSS Inc., Chicago, IL, USA). Descriptive statistics for our samples’ predisposing, enabling, and need characteristics were calculated. The χ^2^ analyses were used to assess differences in background characteristics in HIV testing. In all analyses, the level of significance was set at p < 0.05 (two-tailed). To determine which factors are most strongly associated with HIV testing among young MA users, a multivariate logistic regression model was applied. All covariates were simultaneously entered into the multiple regression models. The odds ratios (ORs) were estimated to assess the strength of the associations and the 95% confidence intervals (CIs) were used for significance testing. The multicollinearity of the variables was checked by examining the variance of inflation factors, which was < 2.0. The logistic regression model was fitted by considering whether the respondents had been tested for HIV or not, with responses dichotomized by assigning “1” for tested and “0” if otherwise. This study calculated the weights as the inverse of the participant’s personal network size. However, no difference was found in the results compared with the unweighted analytical methods. Therefore, this study did not use weighted RDS analysis for the data similar to previous study [36].

## Results

### Descriptive statistics

In terms of predisposing factors, the following characteristics were observed for the sample. The mean age of participants was 21.2 (SD 1.8) years. The highest proportion of the 776 young MA users interviewed belonged to the Shan ethnic group (39.3%) and more than three-fourths (79.1%) were aged between 20-24 years (Table 1). The majority of the respondents reported as being single (85.1%), with the remainder reporting as currently married (11.7%), or separated/divorced/widowed (3.2%). Regarding educational status, 51.3% reported having a secondary level of education while 29.5% reported having higher education. The majority of respondents (61.5%) were currently living alone or with friends. The mean age of participants those who had ever had been tested an HIV was 21.6 (SD 1.8). The rate of HIV testing was 14.7% among both males and females.

**Table 1:** Predisposing, enabling, and need factors according to HIV testing among young (18–24 years) MA users in Muse, Myanmar (N=776)

**Characteristics**	**All subjects (N=776)**	**Subjects who received HIV testing**		
	**N/Mean**	**Yes (n=114)**	**No (n=662)**	**P-value**
	**(Per cent/SD)**	**n/mean (per cent/SD)**	**n/mean (per cent/SD)**	
***Predisposing factors***				
**Age, years**	21.2 (1.8)	21.6 (1.8)	21.1 (1.8)	
**Sex**				< 0.001
Male	455 (58.6)	15 (3.3)	440 (96.7)	
Female	321 (41.4)	99 (30.8)	222 (69.2)	
**Education**				0.008
Primary and below	149 (19.2)	10 (6.7)	139 (93.3)	
Secondary	398 (51.3)	68 (17.1)	330 (82.9)	
Higher	229 (29.5)	36 (15.7)	193 (84.3)	
**Marital Status**				0.502
Single	660 (85.1)	93 (14.1)	567 (85.9)	
Currently married	91 (11.7)	17 (18.7)	74 (81.3)	
Separated/divorced/widowed	25 (3.2)	4 (16.0)	21 (84.0)	
**Ethnicity**				0.132
Shan	305 (39.3)	42 (13.8)	263 (86.2)	
Kachin	183 (23.6)	22 (12.0)	161 (88.0)	
Burma	138 (17.8)	19 (13.8)	119 (86.2)	
Others	150 (19.3)	31 (20.7)	119 (79.3)	
**Currently living with**				0.006
Parents/relatives	195 (25.1)	25 (12.8)	170 (87.2)	
Wife/sexual partner/husband	104 (13.4)	26 (25.0)	78 (75.0)	
Alone/friends	477 (61.5)	63 (13.2)	414 (86.8)	
***Enabling factors***				
**Occupation**				< 0.001
Unemployed	325 (41.9)	23 (7.1)	302 (92.9)	
Employed	451 (58.1)	91 (20.2)	360 (79.8)	
**Migrant (in-country migration)**				0.500
No	397 (51.2)	55 (13.9)	342 (86.1)	
Yes	379 (48.8)	59 (15.6)	320 (84.4)	
**Ever visit/meet NGO clinics or workers**				< 0.001
No	636 (82.0)	42 (6.6)	594 (93.4)	
Yes	140 (18.0)	72 (51.4)	68 (48.6)	
***Need factors***				
**Perceived HIV risk**				0.780
No	481 (62.0)	72 (15.0)	409 (85.0)	
Yes	295 (28.0)	42 (14.2)	253 (85.8)	
**Ever been diagnosed with an STI**				< 0.001
No	514 (66.2)	48 (9.3)	466 (90.7)	
Yes	262 (33.8)	66 (25.2)	196 (74.8)	
**Ever accessed drug treatment**				0.008
No	611 (78.7)	79 (12.9)	532 (87.1)	
Yes	165 (21.3)	35 (21.2)	130 (78.8)	
**Ever shared injection equipment**				0.002
No	654 (84.3)	85 (13.0)	569 (87.0)	
Yes	122 (15.7)	29 (23.8)	93 (76.2)	
**Used heroin in the past 6 months**				0.020
No	560 (72.2)	72 (12.9)	488 (87.1)	
Yes	216 (27.8)	42 (19.4)	174 (80.6)	
**Ever drank alcohol**				0.001
No	93 (12.0)	3 (3.2)	90 (96.8)	
Yes	683 (88.0)	111 (16.3)	572 (83.7)	
**Ever wanted to receive help to stop drug use**				< 0.001
No	584 (75.3)	53 (9.1)	531 (90.9)	
Yes	192 (24.7)	61 (31.8)	131 (68.2)	
Ever convicted of a drug related offence*				< 0.001
No	723 (97.7)	88 (12.2)	635 (87.8)	
Yes	17 (2.3)	10 (58.8)	7 (41.2)	

SD, Standard Deviation. *36 participants did not response to this question.

For the enabling factors, more than half of the respondents (58.1%) were employed, 48.8% were migrant, and 18.0% reported having ever visited NGO clinics or met NGO workers (Table 1). Finally, in terms of need factors, the data showed that a substantial portion of young MA users (33.8%) reported that they had ever been diagnosed with an STI. Among the participants, approximately 28.0% had used heroin in the past six months, 16.0% shared injection equipment and 88.0% reported that they had ever drunk alcohol. Furthermore, 21.3% had ever accessed drug treatment, 25.0% had ever wanted to receive help to stop drug use and 2.3% had ever been convicted for a drug related offence (Table 1).

The bivariate analyses revealed several significant differences in HIV testing across the various predisposing, enabling, and need factors (Table 1). Specifically, MA users aged 20-24 years were more likely to report HIV testing. Reports of HIV testing were also significantly more frequent among female MA users, MA users who had completed secondary education, and those who currently live with their spouse/sexual partner.

Similarly, a significantly higher proportion of the participants who were employed reported HIV testing than participants who were unemployed (Table 1). Reports of HIV testing were significantly more frequent among those respondents who had ever visited NGO clinics or met NGO workers, who had ever been diagnosed with an STI, accessed drug treatment, or who had ever shared injecting equipment. Higher prevalence of HIV testing was also identified among those respondents who had taken heroin in the past six months and who had ever drunk alcohol, as well as among those MA users who had ever wanted to receive help to stop drug use or who had ever been convicted for drug related offences.

### Multivariate analysis

Adjusted for other variables in the model, significant positive predictors of HIV testing included the predisposing factors of being a young female MA user (AOR 27.02; 95% CI 11.44 to 63.83); having higher education (AOR 3.96; 95% CI 1.20 to 13.02); and currently living with one’s spouse/sexual partner (AOR 3.92; 95% CI 1.06 to 14.56; Table 2). Significant enabling factors included being employed (AOR 2.80; 95% CI 1.17 to 6.72) and having ever visited NGO clinics or met NGO workers (AOR 16.95; 95% CI 7.71 to 37.26). Significant need factors were having ever been diagnosed with an STI (AOR 4.83; 95% CI 2.31 to 10.13) and having ever wanted to receive help to stop drug use (AOR 4.96; 95% CI 2.23 to 11.05; Table 2).

**Table 2:** Adjusted odds ratios for associations between predisposing, enabling, and need factors and HIV testing among young MA drug users (18–24 years) in Muse, Myanmar ( N=776)

**Characteristics**	**Received HIV testing**		**P-value**
	**AOR**^**†**^	**AOR**^**†**^**(95% CI)**	
***Predisposing factors***			
**Age, years**	1.01	0.81-1.26	0.958
**Sex**			
Male	1.00	--	
Female	27.02	11.44-63.83	< 0.001
**Education**			
Primary	1.00	--	
Secondary	1.31	0.47-3.65	0.609
Higher	3.96	1.20-13.02	0.024
**Marital Status**			
Single	1.00	--	
Currently married	0.42	0.13-1.39	0.158
Separated/divorced/widowed	1.42	0.32-6.33	0.643
**Ethnicity**			
Shan	1.00	--	
Kachin	0.43	0.16-1.14	0.089
Burma	0.51	0.19-1.39	0.189
Others	0.30	0.10-0.90	0.032
**Currently living with**			
Parents/relatives	1.00	--	
Wife/sexual partner/husband	3.92	1.06-14.56	0.041
Alone/friends	1.23	0.41-3.67	0.708
***Enabling factors***			
**Occupation**			
Unemployed	1.00	--	
Employed	2.80	1.17-6.72	0.021
**Migrant (in-country migration)**			
No	1.00	--	
Yes	1.48	0.56-3.92	0.428
**Ever visit/meet NGO clinics and workers**			
No	1.00	--	
Yes	16.95	7.71-37.26	<0.001
***Need factors***			
**Perceived HIV risk**			
No	1.00	--	
Yes	1.13	0.56-2.28	0.736
**Ever been diagnosed with an STI**			
No	1.00	--	
Yes	4.83	2.31-10.13	<0.001
**Ever accessed drug treatment**			
No	1.00	--	
Yes	0.76	0.28-2.03	0.578
**Ever shared injection equipment**			
No	1.00	--	
Yes	2.30	0.69-7.65	0.173
**Used heroin in the past 6 months**			
No	1.00	--	
Yes	0.49	0.16-1.48	0.208
**Ever wanted to receive help to stop drug use**			
No	1.00	--	
Yes	4.96	2.23-11.05	<0.001
**Ever convicted of a drug related offence**			
No	1.00	--	
Yes	3.48	0.59-20.72	0.170

AOR, adjusted odd ratio; CI, confidence interval. ^†^All the variables were simultaneously entered in the models.

## Discussion

To the best of our knowledge, this is the first study to identify the factors associated with uptake of HIV testing among young MA users in Muse city, Myanmar, where use of MA has sharply risen over the past few decades along with increasing numbers of HIV-infected individuals. Findings from this study indicate that only about one third of young MA users had ever been tested for HIV. This percentage is much lower compared with the findings from another recent study conducted in Lashio, Myanmar, which found the HIV testing rate among adult injecting drug users [IDUs] and non-injecting drug users [NIDUs] to be 76.6% and 45.7%, respectively [8]. The important implication of our study finding is that there is a need to further invest HIV testing and prevention services, particularly among young MA users in Muse city, Myanmar. Without this, the low HIV testing rate may lead to worsening of future HIV epidemics and the spread of other STIs.

The study results revealed that the predisposing factors of gender, education and current living status were associated with HIV testing. HIV testing rates were found to be much lower in males than in females. Similar to our study result, females HIV testing rate was higher than males (50% versus 42%) among adults aged 19–35 years living in Peri-urban communities of Thailand [37]. In contrary, in another study in Thailand, significantly higher proportion of male IDUs had undergone HIV testing than those of female users [38]. The large gap in HIV testing rates between male and female MA users in this study may be partly explained by females having more access to voluntary counseling and testing services during their antenatal period than men do in general [39]. Social and cultural determinants between genders also act as a barrier to HIV testing, since men might be seen as “weak” or “unmanly” if they seek medical care or consult with health professionals [40]. Men’s poor access to HIV testing can also consequently affect their partners, resulting in negative health outcomes [41].

Higher educational status was found to be an important factor associated with HIV testing among young MA users in this study. The probability of undergoing HIV testing increased with increasing educational level in multivariate analyses. This finding is supported by the literature. A study from India found that drug users who had a higher level of education were four times more likely to have been tested for HIV compared with illiterates [42]. This finding suggests that prevention programs should focus greater attention on less educated young MA users to promote the uptake of HIV testing.

The findings also indicated that living with one’s spouse or sexual partner increased the chance of young MA users having had HIV testing. Decreased testing among MA users living with family may reflect reticence to disclose this issue to their parents and relatives, due to barriers such as stigma towards drug addicts, fear of being known as a drug user, and fear of being found out as engaging in sexual activities [43-45]. Interventions therefore need to encourage young methamphetamine users to get tested for HIV, without having to disclose their sexual activity to their parents.

The enabling factors of occupation and having ever visited NGO clinics or met NGO workers were also associated with HIV testing in this study. Young MA users who had a regular job were more likely to receive HIV testing. One possible explanation may be that in recent years the Myanmar National AIDS Program has included the workplace as a priority setting for intervention/prevention efforts [46]. Thus, methamphetamine users who have regular jobs may be more likely to receive HIV tests through such programs.

Young methamphetamine drug users were also more likely to have had HIV testing if they had ever visited NGO clinics or met NGO workers. Recently, many national and international NGOs have been providing a variety of HIV testing services in Myanmar, including referrals and pretest and post-test counseling [33]. With adequate counseling from NGOs, methamphetamine users may become more aware of the possible benefits of taking an HIV test.

Finally, the need factors of having ever been diagnosed with an STI, having ever accessed drug treatment and having ever wanted to receive help to stop drug use were found to be associated with HIV testing. This study found a positive attitude to HIV testing among those respondents who had ever been diagnosed with an STI. There is substantial evidence that individuals who are infected with STIs have an increased risk of acquiring HIV infection through sexual transmission of the virus. For example, it has been found that people who have an STI have a two to five times higher chance of acquiring the HIV virus sexually than uninfected persons [47]. This is because STIs can cause small lesions on genital skin and increased inflammation in the genital tract, which can facilitate the entry of HIV. Effective STI screening, treatment and prevention is an essential component of comprehensive prevention of sexually transmitted HIV. Therefore, the findings of this study suggest that interventions should be implemented to encourage young MA users who have been diagnosed with STIs in particular to get tested for HIV. Integrating HIV testing into STI treatment programs may be an effective way of increasing uptake of HIV testing among young MA users.

The findings also indicate that respondents who had ever wanted to receive help to stop drug use were more likely to receive HIV testing. This may be because drug users who stop using drugs are more prone to start feeling that they could be at risk of HIV due to their risky drug use and sexual behaviors [45], ultimately prompting them to seek HIV counseling and to have their HIV status tested.

This study has some limitations to be considered in the interpretation of study findings. First, HIV testing was self-reported by MA users. However, the level of such reporting can be maximized by the use of CASI compared to other methods [18,48]. Second, as this study is cross-sectional in design, the study cannot evaluate the causality of reported associations, and a further longitudinal study would be necessary to establish directionality. Third, a large percentage of the participants had higher than secondary school education and good access to health care services. This implies that participants who received a high school or college education were oversampled in the RDS sample. This might reflect the application of the CASI method for conducting interviews using RDS. Future studies, especially qualitative studies, are needed to explore the reasons for the non-random selection of participants while using computer surveys in a developing country setting.

Finally, this study was conducted in one area of Myanmar. The young drug users’ behaviors and characteristic that were reported in this study may not be similar to those of young drug users from other parts of Myanmar. Therefore, the generalizability of the results may be limited. Despite such limitations, our results present a first look at HIV testing behaviors among young methamphetamine users in Muse, Myanmar. It may have important implications for establishing a better understanding of the risk factors for HIV testing, thereby helping us to develop new prevention strategies.

## Conclusions

In spite of increasing the service delivery of HIV testing, rates of uptake among young MA users remain very low in Muse city, Myanmar. Our result suggests the importance of further investment in HIV testing and prevention services along with research activities in Muse city, Myanmar. In our study, significant positive predictors of HIV testing included the predisposing factors of being a female MA user, having higher education, and currently living with one’s spouse/sexual partner. Similarly, significant enabling factors included being employed and having ever visited NGO clinics or met NGO workers. Significant need factors were having ever been diagnosed with an STI and having ever wanted to receive help to stop drug use. Participants who had ever accessed drug treatment were less likely to receive HIV testing, however. Integrating HIV testing into STI treatment programs, alongside general expansion of HIV testing services, may be effective in increasing HIV testing uptake among young MA users in Muse city, Myanmar.

## Abbreviations

ATS: Amphetamine type stimulant; MA: Methamphetamine; MSM: Men who have sex with men; CASI: Computer- assisted survey instrument; RDS: Respondent-driven sampling; OR: Odds ratio; AOR: Adjusted odds ratio; CI: Confidence intervals; SPSS: Statistical package for the social sciences; NGOs: Non-governmental organizations; IDUs: Injecting drug users; NIDUs: Non-injecting drug users; STI: Sexually transmitted infections.

## Competing interests

The authors declare that they have no competing interests.

## Authors’ contributions

YMS, KCP, KMW, ST, and TNS designed the study. YMS, TNS, NPEK, NC and ST participated in the data collection and drafted the manuscript. YMS and TNS performed the analyses. KCP, TNS, JEC and YMS edited the draft of the manuscript. All the authors critically reviewed and approved the final manuscript.

## Pre-publication history

The pre-publication history for this paper can be accessed here:

http://www.biomedcentral.com/1471-2458/14/735/prepub
